# Relationship between personality types, work addiction, and quality of work life among nurses and healthcare staff in Iran

**DOI:** 10.1038/s41598-025-23148-y

**Published:** 2025-11-11

**Authors:** Mahtab Fathi, Nafiseh Kananifar, Sayad Hamid Atashpour, Fatemeh Bardideh, Seyed Salar Atashpour

**Affiliations:** 1 Faculty of Humanities, Islamic Azad University of Khomeinishahr, Isfahan, Iran; 2 Department of Social Sciences, University of Stavanger, Stavanger, Norway; 3 Lab for Biopsychosocial Personality Research (BPS-PR), International Network for Well-Being, Stavanger, Norway; 4 Department of Social Sciences, Promotion of Health and Innovation for Well- Being (PHI-WELL), University of Stavanger, Stavanger, Norway; 5 Department of Psychology, Islamic Azad University, Isfahan, Branch, Isfahan, Khorasgan Iran; 6 Department of Counselling and Psychology, Islamic Azad University, Kish International, Iran

**Keywords:** Personality types, Work addiction, Quality of work life, Healthcare staff, Burnout, Occupational health, Psychology, Quality of life

## Abstract

Work addiction is an emerging concern that adversely affects the well-being of healthcare professionals. This study examines the relationship between personality types and work addiction, and how these factors influence the quality of work life among nurses and healthcare staff in Iran. This research examines how personality types, particularly Types A and D, contribute to the development of work addiction and its impact on the overall quality of work life. A cross-sectional study was conducted with participants selected through stratified random sampling. Data were collected using standardized questionnaires that assessed personality type, work addiction, and quality of work life. Statistical analyses, including correlation and regression analyses, were employed to determine the relationships between variables. The findings revealed that Type A individuals exhibited a significant positive correlation with work addiction, while Type D individuals also showed a significant association. Conversely, Type B individuals demonstrated lower tendencies toward work addiction. Moreover, increased work addiction was significantly related to reduced job satisfaction and heightened levels of burnout. The study underscores the importance of implementing targeted interventions—such as stress management programs, flexible work hours, and tailored mental health support—to mitigate work addiction and enhance the quality of work life. However, the cross-sectional design and the specific sample limit the generalizability of the findings. These results provide valuable insights for healthcare organizations seeking to improve occupational health and staff well-being.

## Introduction

The quality of work life (QWL) profoundly shapes the mental health, productivity, and job satisfaction of healthcare professionals, particularly in high-stress environments like hospitals^1^. In Iran’s healthcare sector, nurses and staff face relentless pressures—long shifts, emotional demands, and heavy workloads—that threaten their well-being and performance^2,3^. Enhancing QWL boosts employee motivation and elevates patient care quality, making it a critical priority for healthcare organizations^3,4^. Individual factors, such as personality traits, play a pivotal role in how these professionals navigate workplace challenges^5^.

Personality types shape how healthcare workers respond to stress. Type A individuals, driven by competitiveness, may excel under pressure but risk overworking, while Type D personalities, prone to negative emotions and social inhibition, face heightened burnout^6,7^. These traits can ignite work addiction—an obsessive compulsion to overwork—that disrupts work-life balance and erodes QWL^8,9^. Work addiction, prevalent in healthcare due to demanding schedules, is linked to burnout and reduced job satisfaction, threatening staff and organizational outcomes^10^.

## Theoretical framework

This study is grounded in the Big Five personality model and the Job Demands-Resources (JD-R) model. The Big Five model categorizes personality traits into five dimensions: extraversion, agreeableness, conscientiousness, neuroticism, and openness to experience^11^. Conscientiousness, marked by diligence and perfectionism, can fuel work addiction, while neuroticism increases burnout risk^12^. Type A individuals (competitive) may overwork due to achievement-driven motives, whereas Type D individuals (negative, withdrawn) might use work to escape anxiety^6,13^. The JD-R model posits that job demands (e.g., high workloads, emotional strain) exacerbate stress, while resources (e.g., organizational support, autonomy) enhance motivation^14^. High demands may push Type A individuals toward work addiction, as they thrive in pressure but risk exhaustion, while limited resources amplify stress for Type D personalities, reducing QWL^15^. These frameworks guide the study’s exploration of personality and workplace dynamics in Iranian hospitals.

The conceptual model of this study integrates the Big Five and JD-R frameworks to elucidate the pathways linking personality types, work addiction, and QWL among Iranian healthcare workers. Personality traits, particularly conscientiousness (aligned with Type A) and neuroticism (aligned with Type D), are hypothesized to ignite a cascade of work addiction by amplifying responses to workplace pressures. For instance, Type A individuals, driven by ambition and time urgency, may overcommit to tasks under high job demands, such as intense patient care or administrative burdens prevalent in Iran’s hospitals, leading to compulsive overwork^12,14^. Conversely, Type D individuals, with their propensity for negative affectivity, may channel anxiety into excessive work focus, particularly when resources like peer support or autonomy are scarce, exacerbating stress and diminishing QWL^7,16^. The JD-R model frames work addiction as a mediator, where high demands (e.g., long shifts, emotional labor) intensify the link between personality and work addiction, while resources (e.g., supportive leadership, flexible scheduling) mitigate its impact on QWL^17,18^. This model posits that personality influences work addiction directly and QWL indirectly through this mediating pathway, as supported by research on personality and occupational health outcomes^19^. By testing this model, the study illuminates how individual traits and workplace factors interplay to shape mental health and performance in Iran’s high-pressure healthcare context, offering a blueprint for targeted interventions to enhance employee well-being.

### Literature review

This section synthesizes the literature on the relationships between personality types, work addiction, and quality of work life (QWL) among healthcare professionals, with a focus on high-pressure settings like Iran’s hospitals. Drawing on the Big Five personality model and the Job Demands-Resources (JD-R) model, the review examines these relationships one by one, culminating in hypotheses that guide the current study.

## Personality types and work addiction

Personality traits profoundly influence work behaviors, particularly in demanding healthcare environments^19,20^. The Big Five model highlights conscientiousness and neuroticism as key drivers of work addiction^17,21,22^. Conscientious individuals, often aligned with Type A personalities (competitive, time-urgent), exhibit a relentless drive for achievement that can propel them toward compulsive work habits^23,24^. For instance, Type A nurses may overcommit to patient care tasks, increasing their risk of work addiction^25,26^. Conversely, Type D personalities, characterized by neuroticism and social inhibition, may turn to excessive work as a coping mechanism for negative emotions, particularly under stress^27–29^. Studies in healthcare settings show that Type D individuals are prone to work addiction when facing emotional demands, such as patient distress^30,31^. The JD-R model suggests that high job demands amplify these tendencies, especially for Type A individuals, while limited resources exacerbate work addiction in Type D individuals^32,33^.

### Hypothesis 1

There is a significant relationship between personality types and work addiction among Iranian healthcare workers.

## Personality types and the quality of work life

Personality traits significantly influence how individuals experience their work environment and manage occupational stressors, which directly affects their quality of work life (QWL)^34^. Healthcare workers with Type A personality—characterized by competitiveness and urgency—often experience increased tension and dissatisfaction in high-demand settings, which can reduce QWL^35^. Similarly, Type D personality, associated with negative affectivity and social inhibition, has been linked to greater psychological distress, lower job satisfaction, and diminished well-being in clinical environments^36,37^. In contrast, individuals with more adaptive personality traits may demonstrate better coping strategies, which contribute to higher QWL. These findings suggest that personality types can act as predictors of how healthcare workers perceive and manage work-related challenges, shaping their overall job satisfaction and psychological resilience^38,39^.

### Hypothesis 2

There is a significant relationship between personality types and the quality of work life among Iranian healthcare workers.

## Work addiction and quality of work life

Work addiction, often termed workaholism, involves a compulsive drive to engage in excessive work, leading to detrimental outcomes like burnout, fatigue, emotional exhaustion, depersonalization, and poor work-life balance—all of which undermine quality of work life (QWL)^40^. In high-stress healthcare environments, such as hospitals with limited recovery time, work-addicted staff frequently experience heightened mental health issues, reduced job satisfaction, and strained workplace relationships^41^. These effects are particularly acute in Iranian healthcare settings, where heavy patient loads and resource constraints amplify chronic stress and dissatisfaction^42^. Empirical evidence reinforces this link. Among Iranian nurses, 13.77% were identified as workaholics, with the condition associated with sleep disturbances—like difficulty initiating sleep and extreme daytime sleepiness—and depression, signaling a clear decline in QWL^43^. Similarly, research on Brazilian physicians showed that elevated work addiction correlates with lower overall quality of life, significantly reducing the odds of high QWL^44^. While not Iran-specific, these patterns likely extend to Iranian workers due to comparable workplace pressures, such as demanding shifts and high-stakes patient care.

### Hypothesis 3

There is a significant relationship between work addiction and the quality of work life among Iranian healthcare workers.

Examining the relationship between personality, workaholism, and quality of work life among healthcare employees is of special importance. Employees in this sector, due to constant exposure to job pressures, long shifts, and chronic stress, are at risk of negative consequences resulting from workaholism and a decline in work-life quality (Maisonneuve et al., 2023). Although numerous studies have examined these variables separately, few have focused on the direct relationship between these factors among healthcare workers (Gümüş et al., 2021). Previous research has primarily been conducted in non-medical settings and has provided limited information on how personality affects work-life quality and work addiction in this specific group. The present study examines the direct relationship between personality traits, workaholism, and quality of work life among healthcare employees, addressing the existing research gaps in this area. The research questions are as follows:

1. Is there a significant relationship between personality types and work addiction among Iranian healthcare workers?
2. Is there a significant relationship between personality types and the quality of work life among Iranian healthcare workers?
3. Is there a significant relationship between work addiction and the quality of work life among Iranian healthcare workers?

By answering these questions, this study sheds light on the interaction between personality types, work addiction, and quality of work life in healthcare settings.

## Methods

This study was approved by the Ethics Committee of the University of Isfahan (Khorasgan) by the Declaration of Helsinki (approval no. KH-2020-089). This research involving human participants was conducted in accordance with the relevant guidelines and regulations, including the Declaration of Helsinki. Informed consent was obtained from all the participants before they participated in the study. To ensure confidentiality, all personal data were anonymized to protect the privacy of the participants.

The descriptive-correlational method was chosen for this study to investigate the relationships between personality traits, workaholism, and quality of work life among healthcare staff. This approach allows for the simultaneous examination of multiple variables and helps researchers identify patterns of association without direct intervention^45^. It is commonly used in industrial and organizational psychology to assess how individual characteristics influence job-related outcomes^46^. Moreover, applying this method in workplace studies provides valuable insights into the psychological factors affecting employee performance quality.

### Statistical population

The statistical population of this study consisted of all nurses and staff employed in five medical units, with a total population of 260 individuals. These individuals were selected based on their role in direct patient care, which increased the relevance of investigating work addiction and quality of work life within this group. To ensure a representative sample, five hospitals with varying characteristics in service levels (general, specialized, and super-specialized hospitals), patient volume, and workload were selected. This approach aligns with standard research guidelines, which suggest that studies examining the occupational and psychological characteristics of healthcare staff should include hospitals with diverse conditions to improve result generalizability^47^.

## Hospital selection criteria

- **Diversity in hospital type**: Inclusion of general, specialized, and super-specialized hospitals to enhance generalizability.
- **Variation in workload**: Selection of hospitals with differing patient volumes and workload intensities.
- **Access to diverse healthcare professionals**: Ensuring participation from different staff categories, including nurses, doctors, and support personnel.
- **Feasibility of research collaboration**: Preference for hospitals where data collection and researcher collaboration were more accessible.

Once these five hospitals were selected, stratified random sampling was employed. This method was chosen because in studies where the population consists of multiple subgroups with distinct characteristics (e.g., nurses, doctors, and support staff), simple random sampling can lead to disproportionate representation of certain groups^48^.

## Sampling

From a total of 32 medical units, five units were randomly selected, and all nurses and staff (260 individuals) within these units constituted the population for this study. A sample of 152 individuals was established using the Jessi–Morgan table to determine the sample size. The sampling method was stratified random sampling, ensuring the proportional representation of nurses and staff in each medical unit. The sample size was determined using the Krejcie and Morgan Table (1970), a widely recognized method for calculating appropriate sample sizes in social and medical sciences research. According to this table, for a total population of 260 individuals, a minimum sample of 152 participants ensures a 95% confidence level with an acceptable margin of error. Additionally, previous studies indicate that for correlational research, a sample size of 10 to 15 participants per independent variable is typically sufficient^49^. Given the number of independent variables examined in this study, the selected sample size aligns with these methodological standards. Furthermore, conducting research in real workplace environments presented challenges in participant accessibility. The demanding schedules of healthcare professionals and the potential for participant dropout necessitated a practical and manageable sample size^50^. To enhance generalizability, response rates were carefully monitored, and supplementary analyses were conducted to ensure the results were free from sampling bias.

### Control variables

In order to improve the accuracy of the results, three control variables were included in the statistical analyses: age, education level, and work experience. The participants’ work experience was categorized into three groups: 1–5 years (32.5%), 6–10 years (33.1%), and more than 10 years (31.1%). Regarding education level, 19.2% of participants had less than a diploma, 50.3% had a diploma, and 30.5% held an education above diploma level. These variables were collected as part of the demographic questionnaire and were included in the regression models to control for their possible effects on personality type, work addiction, and quality of work life.

### Data collection

#### Method

Data were collected through face-to-face questionnaires, a method selected for several reasons: Enhanced accuracy and reduced response error. Face-to-face interactions enable participants to seek clarification on ambiguous questions, thereby improving the reliability and quality of the data collected^51^. Increased response rate: In comparison to online or postal surveys, in-person distribution reduces non-response rates and fosters greater participation^52^. Controlled response conditions: Administering the survey within the hospital setting ensured that participants responded under standardized conditions, thereby minimizing external distractions and enhancing data validity.

### Type of research

Cross-Sectional This study employs a cross-sectional design, wherein data were collected at a single point in time. This methodological approach was selected for several reasons: Simultaneous evaluation of variable relationships: Cross-sectional studies facilitate the examination of statistical associations between variables without necessitating long-term follow-up^53^. Suitability for workplace settings: Given the demanding nature of healthcare occupations, this method permitted efficient data collection without disrupting staff schedules^54^. Resource constraints: Longitudinal studies require substantial time and financial resources, which were not feasible for this research.

### Participant recruitment and access

The research team collaborated with hospital management to recruit participants, securing the necessary permissions before visiting various hospital departments. Researchers offered comprehensive explanations regarding the study, and staff members who voluntarily consented were included in the research. This approach facilitated an increase in participation rates by ensuring that staff comprehended the study’s purpose and significance and the utilization of professional hospital networks and administrative coordination to access a representative sample.

### Data collection tools

The following standardized questionnaires containing 144 items were used for data collection:

- **Walton’s quality of work life questionnaire**: This questionnaire includes 45 questions, scored on a Likert scale, and assesses eight different dimensions that contribute to overall job satisfaction and well-being in the workplace, including both internal (growth and development opportunities) and external (workplace safety and fair pay) factors. It evaluates eight dimensions: fair payment, legality, growth and security opportunities, social dependency, personal development, the general work atmosphere, environmental safety, and social integration. Two sample questions from the questionnaire include: “My supervisors appropriately acknowledge my efforts and hard work” and “Supervisors act upon the constructive criticisms of their subordinates.”
- **Work addiction questionnaire (karimi & atashpour**,** 2001)**: This questionnaire consists of 30 questions using a five-point Likert scale. A score above 135 indicated severe work addiction, a score between 105 and 134 indicated mild work addiction, and a score below 105 indicated no work addiction. Two sample questions from the questionnaire include: “I prefer to complete tasks independently rather than request assistance from others” and “When awaiting tasks or when their completion is delayed, I become agitated and frustrated with myself.”

### Personality type questionnaire

**Type a and b personality**^55^: This section includes 25 dichotomous questions. The Type A and B Personality Questionnaire (Rathus, 1999) is designed to assess individuals’ personality traits on a spectrum between Types A and B. Type A personality is typically characterized by competitiveness, impatience, a high sense of urgency, and a tendency toward hostility and stress. Individuals with Type A traits are often seen as ambitious, goal-oriented, and driven but may also be prone to stress-related health issues due to their intense nature. Type B personality, on the other hand, is associated with being more relaxed, patient, and easygoing. Individuals with Type B traits tend to handle stress better, are less focused on competition, and are generally more adaptable to social and work situations. This distinction between Type A and B personalities was developed as part of research on behavioral patterns and their effects on health, especially about stress and cardiovascular risk. Rathus’s questionnaire helps in identifying these personality types to understand their psychological and health implications. This tool is part of Rathus’s broader work on psychology and behavioral patterns, focusing on distinguishing between Type A and Type B personalities. Two sample questions from the questionnaire include: “When I encounter others, I easily establish communication with them” and “I often lose my temper.”

**Type C personality**^56^: This scale includes 30 questions scored using a Likert scale. Type C Personality is characterized by emotional suppression, excessive cooperation, and avoidance of conflict. Individuals with this personality type tend to internalize negative emotions, particularly anger and frustration, to maintain harmony, which can lead to chronic stress. This emotional repression is thought to weaken the immune system and increase vulnerability to illnesses, particularly cancers. The work of these researchers emphasizes the psychoneuroimmunological connection, highlighting how personality traits and emotional regulation can influence health outcomes. Their findings suggest that individuals with Type C personality, often referred to as “cancer-prone,” may experience poorer health outcomes due to their chronic emotional suppression, underlining the need for psychological interventions to improve stress management and emotional expression. Two sample questions from the questionnaire include: “I prefer to complete tasks independently rather than seeking assistance from others,” “When awaiting tasks or experiencing delays in their completion, and I become frustrated and disheartened”.

**Type D personality**^57^: In this study, Type D Personality, as described by Johan Denollet, was evaluated using the DS14 Scale. Type D personality is defined by high levels of negative affectivity, a tendency to frequently experience negative emotions, and social inhibition, where individuals avoid social interactions due to fear of rejection or disapproval. The DS14 Scale includes 14 items divided into two subscales: negative affectivity (seven items) and social inhibition (seven items). Individuals who scored high on both subscales were classified as having Type D personalities. This personality type is strongly associated with negative health outcomes, particularly in patients with cardiovascular disease, where it is associated with a higher risk of poor prognosis and mortality. Denollet’s work highlights the critical need to address emotional well-being and stress management in clinical care to improve health outcomes for individuals with Type D personalities. Two sample questions from the scale include: “I often feel dissatisfied” and “I am usually nervous.”

### Validity and reliability

To determine the validity and reliability, a pilot study was conducted with a separate sample of 30 individuals from the same population before administering the main test. The reliability of the Work Addiction questionnaire was assessed using Cronbach’s alpha coefficient, which was found to be 0.84 for the Quality of Work Life questionnaire and 0.78 for the Work Addiction questionnaire. As the Cronbach’s alpha values for each questionnaire were above 0.7, the reliability of the questionnaires can be considered acceptable. Content validity was used to assess the validity of the questionnaire. The questionnaires were reviewed and approved by supervisors, advisors, and experts in the field, and necessary revisions were made. It is worth noting that the validity and reliability of these questionnaires have been confirmed in previous studies. For the main test’s reliability, the Cronbach’s alpha method was employed, and the Cronbach’s alpha coefficients for the dimensions of the questionnaires were calculated using SPSS software. As seen in Table 1, all coefficients were above 0.7, indicating high reliability of the questionnaires.

**Table 1:** Cronbach’s alpha values for Reliability.

Main Structures	Cronbach’s Alpha	Components	Cronbach’s Alpha
Quality of Work Life	0.82		
Personality Types	0.85	Type A & B	0.77
		Type C	0.87
		Type D	0.82
Work Addiction	0.80		
All Items Combined	0.89		

Table 1 presents the reliability of each questionnaire, measured using Cronbach’s alpha, which is a common statistical test for internal consistency. Reliability above 0.70 is considered acceptable in social sciences research, was considered acceptable. The following key findings were noted:

- Quality of Work Life (α = 0.82) showed high reliability in measuring various dimensions of workplace satisfaction.
- Personality Types (α = 0.85) are broken down into three subcategories, with Types A and B scoring 0.77, Type C scoring 0.87, and Type D scoring 0.82, indicating that the questionnaire reliably measures these constructs.
- Work Addiction (α = 0.80) confirmed that the items effectively assessed work addiction among participants.

The overall reliability score for the entire questionnaire was 0.89, indicating very high internal consistency.

## Results

### Descriptive statistics

The information obtained from descriptive statistics indicated that 36.4% of the employees participating in this study had 6–10 years of work experience. Of the participants, 31.1% had more than ten years of work experience. A total of 32.5% had 1–5 years of work experience. Based on the collected data, 21.2% of the participants were addicted to work, while 78.8% were not. The mean score for work addiction among participants was 93.09. Given that a score of 105 is the cutoff point, the level of work addiction among individuals was estimated to be below average, with a standard deviation of 12.8. The distribution is normal.

In this study, participants scored an average of 143.95 on the Quality of Work Life Questionnaire, which was slightly above the average score of 135. The standard deviation of the distribution was 18.5, indicating relatively low skewness and a normal distribution. According to the collected data, the average score of participants for personality types A and B was 12.8, which is approximately equal to the cut-off point of 13. The average score for Type C was 46.14, with a standard deviation of 8.02. For Type D, the average score was 21.15, with a standard deviation of 9.3, which was slightly above the cut-off point of 20.

### Inferential statistics

This study examined the relationships between the variables using Pearson’s correlation and multiple regression models. The results indicate significant correlations between personality types and work addiction and between work addiction and quality of work life.

**Table 2:** The correlation between personality types and work addiction.

	Work Addiction	Type A & B	Type C	Type D
Work Addiction	1			
Type A & B	0.464*	1		
Type C	0.002**	0.092**	1	
Type D	0.352*	0.372*	0.07**	1

*p* < 0.01*, *p* < 0.5**.

Table 2 shows the correlation between personality types and work addiction. Significant correlations were noted for Types A and B (*r* = 0.464, *p* < 0.01), which showed a moderate positive correlation with work addiction, suggesting that individuals with these personality types are more likely to develop work addiction. Type D (*r* = 0.352, *p* < 0.01) also showed a significant positive correlation, implying that individuals with Type D personality traits, known for negative affectivity and social inhibition, are prone to work addiction. Type C (*r* = 0.002, *p* = 0.5) showed no significant relationship with work addiction, indicating that this personality type does not contribute significantly to work addiction. The table confirms that personality traits, especially Types A, B, and D, are predictive of work addiction tendencies among healthcare staff members. This highlights the need for targeted interventions that focus on these personality types to mitigate work addiction and its associated negative effects on job performance and well-being.

**Table 3:** Multiple regression analysis between personality types and quality of work Life.

Model	Predictor Variables Entered into the Model	Beta	t	Significance Level (*p*)	Correlation Coefficient (*r*)	*R*-squared
1	Type A & B	0.042	0.48	0.64	0.097	0.009
	Type C	0.066	0.79	0.43		
	Type D	−0.078	−0.88	0.38		

Table 3 displays the results of the multiple regression analysis between personality types (Types A, B, C, and D) and quality of work life. The correlation coefficient (*r* = 0.097) and R-squared (0.009) indicate that personality types, as a whole, have a weak relationship with the quality of work life among healthcare employees. The beta values show the strength of the influence of each personality type on the quality of work life. However, none of the beta values reached statistical significance, as indicated by the high p values (greater than 0.05). This means that personality types, while important in shaping work behaviors, did not significantly predict the quality of work life in this sample. This finding suggests that while personality traits might play a role in work addiction, their direct impact on the quality of work life is minimal. This may indicate that other factors, such as organizational culture or workload, are more influential in determining employees’ perceptions of their work environments.

**Table 4:** Correlation between work addiction and quality of work Life.

	Work Addiction	Quality of Work Life
Work Addiction	1	
Quality of Work Life	0.027**	1

*p* < 0.01*, *p* < 0.5**.

Table 4 shows the correlation between work addiction and quality of work life. A positive correlation was found between these two variables (*r* = 0.158, *p* = 0.027), which was statistically significant at the 0.05 level. This suggests that as work addiction increases, so does the perceived quality of work life of healthcare employees. This result may seem counterintuitive, but it aligns with findings in other high-stress professions, where individuals with higher work addiction may feel a stronger sense of accomplishment or self-worth derived from their jobs, temporarily improving their perception of work-life quality. However, although the correlation was statistically significant, it was relatively weak. This indicates that work addiction alone does not fully explain the changes in the quality of work life and that other factors are likely to influence this relationship. Further analysis could explore whether factors such as job satisfaction and organizational support mediate this correlation.

## Discussion

Theoretical foundations indicate that personality traits can significantly impact individuals’ work behaviors. Type A individuals are typically competitive, perfectionistic, and seek quick successes. These traits can drive them towards excessive work, as they feel they must always be engaged in professional activities to achieve their goals. On the other hand, Type D individuals, who are prone to experiencing negative emotions and social avoidance, may use work as a way to escape their inner anxieties and worries. In contrast, Type B personalities, with a calmer approach and better work-life balance, are less likely to become workaholics. According to the statistical analyses in Table 3, which show the relationship between personality types and work addiction, it has been determined that Type A and B personalities have a positive and significant relationship with work addiction (*r* = 0.464, *p* < 0.01). This means that individuals with these personality traits are more likely to engage in workaholic behaviors. Additionally, type D personality also has a positive and significant relationship with workaholism (*r* = 0.352, *p* < 0.01), indicating that these individuals, due to their tendency towards negative emotions and social worries, may become excessively immersed in their work. In this context, Type C personality showed no significant relationship with workaholism (*r* = 0.002, *p* = 0.5), indicating that the characteristics of this personality type do not have a noticeable impact on the level of individuals’ engagement with work.

Therefore, the research data indicate that personality types, especially A and D types, have a direct impact on the likelihood of developing work addiction, while other personality traits do not play a significant role in this regard. These findings are consistent with previous research that has shown that individuals with Type A personality, due to their high motivation for success and time pressure, and individuals with Type D personality, due to social anxiety and negativity, are more prone to overwork and work addiction^58,59^. Based on theories related to the quality of work life, workaholism can harm job satisfaction and health. Individuals affected by this issue usually allocate less time for rest, social interactions, and personal well-being improvement, resulting in burnout and decreased job satisfaction. Workaholism may also lead to increased stress and excessive fatigue, which in the long term reduces the quality of work life and job dissatisfaction.

The statistical analyses presented in Table 4, which examine the relationship between workaholism and quality of work life, indicate that the correlation between these two variables is positive and significant but weak (*r* = 0.158, *p* = 0.027). A p-value less than 0.05 indicates the significance of this relationship, but its strength is low. These findings indicate that workaholism is somewhat related to work-life quality, but this relationship is not very strong. It seems that other factors, such as the work environment, the level of organizational support, and the level of job stress, also play an important role in the quality of work life. Therefore, although the results confirm that an increase in workaholism can affect the quality of work life, the intensity of this impact is limited, and other influencing variables should also be considered. The findings of this study are consistent with the results of Sussman^60^, which showed that workaholism is directly related to a decrease in work-life quality, but this relationship is moderated in some work environments with organizational support.

From a theoretical perspective, it may be expected that personality traits have a direct impact on the quality of work life. For example, individuals with Type A personalities may experience job stress due to their tendency towards competitiveness and hard work, which can affect their quality of work life. Similarly, Type D personality, characterized by negative emotions and anxiety, may feel more pressure in the workplace and experience a lower quality of work life. However, it is also possible that environmental factors such as job support, organizational culture, and working conditions have more significance than personality traits in determining the quality of work life. Statistical analyses in Table 4, which show the relationship between personality types and quality of work life, indicate that the overall correlation coefficient is very low (*r* = 0.097) and the R² value is 0.009, which means that only 0.9% of the changes in quality of work life can be explained by personality types. Furthermore, none of the personality types showed a significant impact on work-life quality (all p-values were greater than 0.05). These findings indicate that personality types cannot directly predict work-life quality, and environmental variables likely play a more prominent role in this regard. Consequently, this hypothesis was not confirmed in the present study, and the data indicate that work-life quality is primarily influenced by other factors, such as the work environment and the level of organizational support. >0.05). These findings contradict some previous studies that showed specific personalities (such as Type A and D) can have a significant impact on work-life quality^61^. In contrast, these findings are consistent with the results of the study by Aronsson, Theorell^62^, which showed that work-life quality is more influenced by the work environment, organizational support, and economic conditions than by personality traits.

The results of this study regarding the impact of personality traits on workaholism are consistent with the findings of Andreassen, Pallesen^40^. In this study titled “Workaholism and Personality,” it was found that individuals with Type A personality (competitive and perfectionist) and Type D personality (prone to negative emotions) have the highest tendency towards workaholic behaviors. These results indicate that competitive motivations and stress caused by negative thoughts can drive individuals towards excessive work (Andreassen et al., 2018). The findings of the present study also confirm this relationship; therefore, these two studies are consistent with each other. Additionally, the research by Clark et al. (2020) titled “Personality and Work-life Integration” showed that individuals with competitive and negative personalities are more exposed to job pressures and work dependency. This study emphasizes that personality traits can influence the way job stress is experienced and the level of work addiction^63^. The findings of the present study also confirm this issue and show that Type A and D personalities, due to their specific personality traits, are more prone to behaviors related to work addiction. Therefore, this research is also consistent with the present study.

In the context of the impact of workaholism on quality of work life, the findings of the present study are consistent with the results of the research by Sussman et al. (2011) conducted in a study titled “Workaholism and Quality of Life.” This study showed that workaholism can negatively impact the quality of work life, but the intensity of this impact depends on environmental factors such as the level of organizational support and work culture^60^. The present study also showed that although there is a significant relationship between workaholism and work-life quality, this relationship is relatively weak and is likely influenced by other environmental variables. As a result, this research is consistent with the findings of the present study. The study by Schaufeli et al. (2009) in their research titled “Workaholism and Quality of Work Life” also confirmed that workaholism can lead to increased job stress, burnout, and decreased job satisfaction^64^. The findings of the present study also showed that workaholism can lead to a decrease in the quality of work life, although this effect was relatively weak and indicative of the presence of other influencing factors in this area. Therefore, this study is also consistent with the present research.

In the context of the relationship between personality traits and quality of work life, the findings of the present study are in contrast with the results of the research by Makabe et al. (2015). This study, titled “Personality Type and Quality of Work Life in Engineers,” showed that personality types can directly affect the quality of work life^61^. In the present study, no significant relationship was found between personality types and quality of work life, and the impact of this variable was attributed more to environmental factors. Therefore, this study is not consistent with the present research. In contrast, the findings of the present study are consistent with the results of the research by Aronsson et al. (2017). In this study titled “The Work Environment and Well-being at Work: Employees’ Experiences in the Public Sector,” it was shown that work-life quality is more influenced by the work environment, organizational support, and economic conditions than by personality traits^62^. The findings of the present study also showed that individuals’ personalities do not play a significant role in predicting work-life quality, and environmental factors likely have a stronger impact in this regard. Therefore, this research is consistent with the present study.

## Conclusion

This research was conducted to examine the relationship between personality types, workaholism, and quality of work life among healthcare employees. The findings from the data analysis indicated that the research objectives were largely achieved, but some theoretical expectations did not align with the actual results. The obtained results confirmed that personality types, especially Type A and D personalities, have a significant impact on work addiction. These findings indicate that individuals with a competitive personality (A) and those prone to experiencing negative emotions (D) are more susceptible to work addiction, which aligns with theoretical expectations and previous research. Therefore, this part of the research objectives was fully achieved. In examining the relationship between workaholism and quality of work life, the results showed that this relationship is statistically significant but relatively weak. Although it was expected that workaholism would have a stronger negative impact on the quality of work life, the observed correlation was low. This indicates that, in addition to workaholism, other factors such as organizational support, job stress levels, and working conditions also affect the quality of work life. Therefore, this part of the research only partially confirmed the intended objectives and showed that the quality of work life is influenced by more complex factors. Regarding the relationship between personality types and quality of work life, the research results were contrary to initial expectations. The analyses showed that personality does not have a significant impact on the quality of work life, whereas it was expected that specific personalities (such as A and D) would directly influence it. This result indicates that personality traits alone do not determine the quality of work life and environmental factors play a more prominent role in this regard. Therefore, this part of the research objectives was not achieved, and it showed that more mediating and moderating variables need to be examined in future studies. Overall, this research achieved some of its objectives, particularly regarding the relationship between personality types and workaholism. However, in some cases, the findings indicated that other factors also influence work-life quality, which were not considered within the framework of this study. Therefore, it is suggested that future research examine mediating variables such as organizational culture, social support, and job stress to provide a more accurate understanding of these relationships.

### Theoretical implications

This study adds to the existing literature by clarifying the relationships between personality traits, work addiction, and quality of work life. It does so through the lens of the Big Five personality model and the Job Demands–Resources (JD-R) framework. The significant associations observed between Type A and Type D personality profiles and work addiction reinforce the theoretical relevance of traits such as conscientiousness and neuroticism in the development of compulsive work behaviors^11,60,65^. These results contribute to the JD-R model by illustrating how certain personality traits may function as personal vulnerabilities that intensify the effects of high job demands, particularly in healthcare environments where stress levels are consistently elevated^66^.

The absence of a direct relationship between personality types and quality of work life suggests that environmental and organizational factors may play a critical mediating or moderating role. This finding highlights an important theoretical gap, as traditional personality models often overlook the contextual elements that shape workplace well-being. Future research should seek to expand the JD-R model by integrating variables such as organizational culture, leadership style, and social support^40^. This would allow for a more comprehensive understanding of how personality interacts with the work environment to influence outcomes such as job satisfaction, burnout, and engagement.

#### Practical implications

The findings of this study offer valuable guidance for healthcare administrators, especially within the Iranian healthcare system. The link between specific personality types and work addiction indicates the potential benefits of incorporating personality assessments into staff development or well-being programs. Rather than using such assessments for recruitment purposes, they can serve to identify individuals who may be more vulnerable to overworking. Tailored interventions, including stress reduction workshops, mindfulness-based training, and counseling services, can then be offered to support these employees and reduce the risk of long-term negative outcomes^67^.

In addition to focusing on individual characteristics, organizational improvements are essential. Enhancing workplace resources such as supportive leadership, transparent communication, flexible scheduling, and opportunities for professional growth can significantly improve the quality of work life for healthcare staff^4,40^. These strategies not only protect employee mental health but also contribute to higher levels of job performance and more effective patient care. By addressing both individual and organizational factors, healthcare institutions can create a more sustainable and supportive work environment.

#### Limitations

The results of this study should be interpreted with a number of limitations in mind. First, the results’ applicability of the results to other healthcare settings or industries is limited by the comparatively small sample size (152 participants) and the study’s exclusive focus on five hospitals. Larger and more varied sample sizes should be used in future studies to improve the findings’ generalizability in various work settings. Second, it is impossible to establish causal relationships between the variables because of the study’s cross-sectional design. Longitudinal studies are required to investigate causal links and temporal relationships, even though associations between work addiction, personality, and work-life quality were found. Furthermore, social desirability bias may be introduced by using self-reported data, especially in delicate areas like work addiction. Future research could include objective metrics, like behavioral evaluations or supervisor ratings, to lessen this. Recognizing these limitations strengthens the validity of our findings and aids in directing future studies. Additionally, the impact of personality types was assessed using multiple regression analysis, which may obscure the unique effects of each personality trait. Future studies are recommended to examine personality types individually to better understand their distinct contributions. Recognizing these limitations strengthens the validity of our findings and aids in directing future studies.

#### Disclosure statement

No potential conflict of interest was reported by the author(s).

## Data Availability

The datasets used and/or analysed during the current study are available from the corresponding author upon reasonable request.
